# Osteocyte dysfunction promotes osteoarthritis through MMP13-dependent suppression of subchondral bone homeostasis

**DOI:** 10.1038/s41413-019-0070-y

**Published:** 2019-11-05

**Authors:** Courtney M. Mazur, Jonathon J. Woo, Cristal S. Yee, Aaron J. Fields, Claire Acevedo, Karsyn N. Bailey, Serra Kaya, Tristan W. Fowler, Jeffrey C. Lotz, Alexis Dang, Alfred C. Kuo, Thomas P. Vail, Tamara Alliston

**Affiliations:** 1 Department of Orthopaedic Surgery, University of California, San Francisco, CA 94143 USA; 2 UC Berkeley-UCSF Graduate Program in Bioengineering, San Francisco, CA 94143 USA; 3 Department of Mechanical Engineering, University of Utah, Salt Lake City, UT 84112 USA; 4 San Francisco Veterans Affairs Medical Center, San Francisco, CA 94121 USA

**Keywords:** Bone, Pathogenesis, Bone quality and biomechanics

## Abstract

Osteoarthritis (OA), long considered a primary disorder of articular cartilage, is commonly associated with subchondral bone sclerosis. However, the cellular mechanisms responsible for changes to subchondral bone in OA, and the extent to which these changes are drivers of or a secondary reaction to cartilage degeneration, remain unclear. In knee joints from human patients with end-stage OA, we found evidence of profound defects in osteocyte function. Suppression of osteocyte perilacunar/canalicular remodeling (PLR) was most severe in the medial compartment of OA subchondral bone, with lower protease expression, diminished canalicular networks, and disorganized and hypermineralized extracellular matrix. As a step toward evaluating the causality of PLR suppression in OA, we ablated the PLR enzyme MMP13 in osteocytes while leaving chondrocytic MMP13 intact, using Cre recombinase driven by the 9.6-kb DMP1 promoter. Not only did osteocytic MMP13 deficiency suppress PLR in cortical and subchondral bone, but it also compromised cartilage. Even in the absence of injury, osteocytic MMP13 deficiency was sufficient to reduce cartilage proteoglycan content, change chondrocyte production of collagen II, aggrecan, and MMP13, and increase the incidence of cartilage lesions, consistent with early OA. Thus, in humans and mice, defects in PLR coincide with cartilage defects. Osteocyte-derived MMP13 emerges as a critical regulator of cartilage homeostasis, likely via its effects on PLR. Together, these findings implicate osteocytes in bone-cartilage crosstalk in the joint and suggest a causal role for suppressed perilacunar/canalicular remodeling in osteoarthritis.

## Introduction

Osteoarthritis (OA), the most common chronic joint disease, is a leading cause of pain and disability worldwide.^1^ OA irreversibly damages articular cartilage and the surrounding tissues, compromising joint function and mobility of over 30 million Americans.^2^ Abundant research efforts have investigated cartilage and its interactions with other joint tissues in order to understand the underlying mechanisms of OA initiation and progression.^3–5^ Still, no permanent disease-modifying therapies exist short of joint replacement.

A major question in the field is the extent to which subchondral bone plays a causal role in the pathogenesis of OA. Though much correlative evidence indicates the coordinated degradation of subchondral bone and cartilage,^3,6^ causality is difficult to ascertain because analyses are often conducted on tissues with end-stage disease or in models in which both the bone and cartilage are affected. Recent studies have illuminated that biological^7^ and mechanical^8^ changes to the subchondral bone can precede degradative changes to overlying cartilage. However, the cellular mechanisms responsible for OA-related changes in subchondral bone, and particularly the role of osteocytes, remain unclear.

Recent reports have reinvigorated interest in the active role of osteocytes in remodeling their surrounding bone matrix—a process called perilacunar/canalicular remodeling (PLR).^9–12^ PLR is a dynamic process by which osteocytes secrete matrix metalloproteinases (MMPs),^13–15^ cathepsin K (CatK),^10^ and other enzymes^16,17^ to dynamically resorb and then replace the local bone matrix. PLR maintains bone material properties,^13,18,19^ systemic mineral homeostasis,^10,12^ and the canalicular channels that facilitate osteocyte communication, mechanosensation, and nourishment.^9,20,21^ Several known regulators of bone homeostasis, including TGF-β,^19^ SOST,^17^ parathyroid hormone,^10,22^ and Vitamin D,^23,24^ regulate PLR to support the metabolic and mechanical function of the skeleton. Although PLR is a fundamental mechanism by which osteocytes maintain bone homeostasis, its role in the maintenance of subchondral bone and the progression of joint disease remain unclear.

To elucidate the role of PLR in disease, we previously investigated osteonecrosis, a progressive and severe joint disease in which subchondral bone mechanically fails with painful collapse of the articular surface.^25,26^ We found that glucocorticoids, a major risk factor associated with osteonecrosis,^25^ suppress PLR and cause the same changes in subchondral bone of mice as seen in glucocorticoid-induced human osteonecrosis.^27^ Although suppression of PLR is clearly associated with the degradation of subchondral bone in osteonecrosis, it was not possible to isolate the effects of osteocytes from the systemic influence of glucocorticoids. Given that the health of articular cartilage depends upon subchondral bone for mechanical and vascular support,^28,29^ we hypothesized that signs of PLR dysregulation may also accompany the much more common joint disease, osteoarthritis. We tested this hypothesis by examining specific hallmarks of PLR suppression and their relationship to cartilage degeneration in OA of the human knee. As a step toward evaluating the causality of PLR suppression in joint disease, we evaluated the bone and joint phenotypes of a novel mouse model with ablation of the critical PLR enzyme MMP13 from osteocytes, but not chondrocytes. Together, our results demonstrate the causal role of osteocyte-derived MMP13 in the pathogenesis of OA, suggesting the importance of osteocyte perilacunar/canalicular remodeling for joint homeostasis.

## Results

### Degeneration of the osteocyte lacunocanalicular network in human osteoarthritis

**Fig. 1:**
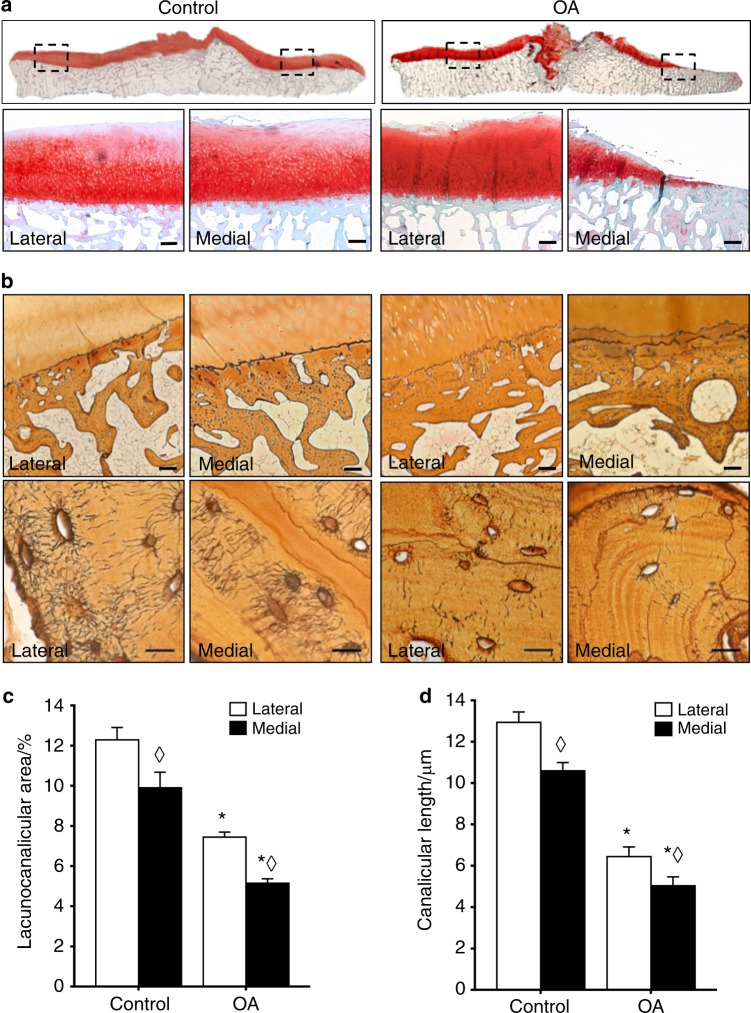
Lacunocanalicular networks are disrupted in human OA subchondral bone. **a** Control cadaveric and OA specimens stained with Safranin-O/Fast Green and imaged at 0.5x (top) or 2x (bottom, scale bars: 400 μm) magnification displayed differences in articular cartilage and subchondral bone morphology on the lateral and medial sides of the tibial plateau. Subsequent analyses compared the indicated regions of interest (black boxes on top row) between control and OA specimens, and between the less affected lateral side with the more severely degraded medial side. **b** These identified regions of interest in Ploton silver-stained sections were evaluated at low (4x, top, scale bars: 200 μm) and high (100x, bottom, scale bars: 20 μm) magnification to visualize the lacunocanalicular network of subchondral bone. **c**, **d** Quantification of the lacunocanalicular area normalized to the bone area (**c**) and canalicular length (**d**) revealed significant OA-dependent reductions in both parameters (*n* = 5). Graphs show mean ± SEM. *P < 0.05 compared with respective regions of control specimens, ^⋄^*P* < 0.05 between regions by Holm–Sidak post hoc tests

To determine if osteocytic PLR is affected by OA in humans, we compared subchondral bone in tibial plateaus from patients with end-stage OA to that of cadaveric donors with no clinical evidence of joint disease. As expected, tibial plateaus from patients with OA had gross degeneration of the articular cartilage (Supplementary Fig. 1a–f) and radiographic evidence of subchondral bone sclerosis, particularly on the medial side of the joint (Supplementary Fig. 1g–i). Histological analysis confirmed the cartilage degeneration and subchondral bone sclerosis in OA specimens (Fig. 1a). Consistent with prior reports,^6^ both the subchondral bone plate and trabeculae were thicker in OA than in controls (Fig. 1a). Relative to the cadaveric controls, OA tibial plateaus showed decreased cartilage thickness, reduced Safranin-O-positive proteoglycan staining in the superficial zone, and cartilage fibrillation (Fig. 1a), resulting in significantly higher OARSI scores (Supplementary Fig. 1b). Although the control cartilage was more intact than the OA cartilage overall, the medial compartment showed more evidence of degeneration in both OA and control specimens. Therefore, the subsequent analyses of subchondral bone compared defined regions of interest between the control and OA specimens, as well as between the medial and lateral side of the same specimen.

While OA-dependent differences in osteoblast and osteoclast function have been described,^3,6^ the effect of OA on osteocytes is not well-defined. Therefore, we evaluated osteocyte PLR in subchondral bone by studying one of its key hallmarks, the lacunocanalicular network (LCN). Silver staining revealed that cadaveric control subchondral bone had both more abundant and apparently longer canalicular projections than the subchondral bone from OA patients (Fig. 1b). The dramatic degeneration of the canalicular network in OA bone was particularly evident in the medial side of the tibial plateau, where cartilage degeneration was most severe. Quantitative analysis revealed significant OA-dependent reductions in the total osteocyte lacunocanalicular area (38%–46%) (Fig. 1c) and canalicular length (51%–54%) (Fig. 1d) relative to cadaveric controls, consistent with this hallmark feature of PLR suppression.^10,13–15,19,27,30^ Therefore, the reduced canalicular length and lacunocanalicular area in OA subchondral bone strongly suggests that PLR is suppressed in OA.

### Collagen disorganization and hypermineralization in human OA subchondral bone

**Fig. 2:**
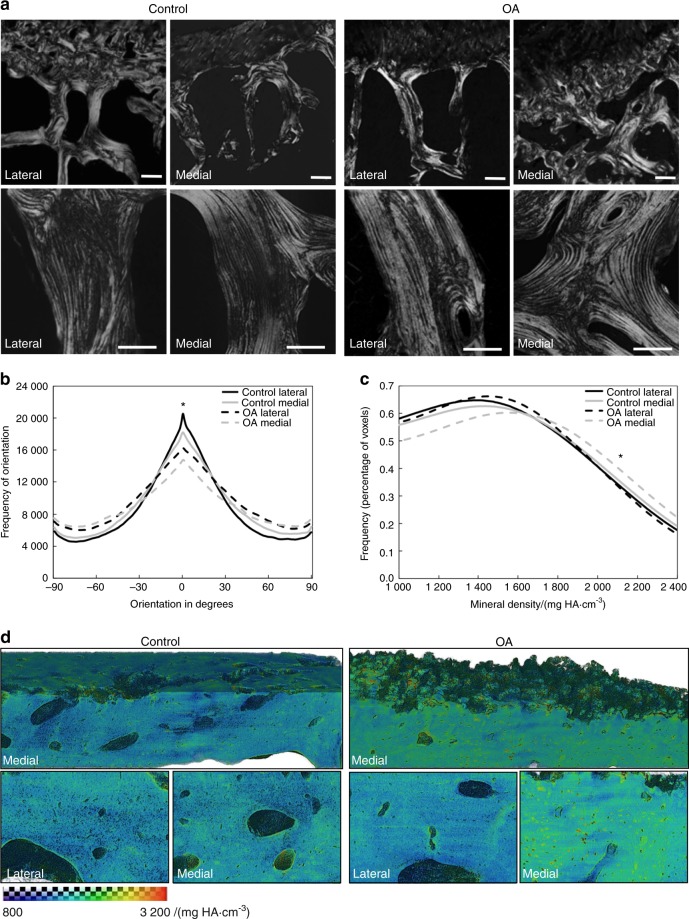
Human OA subchondral bone shows collagen disorganization and hypermineralization. **a** Control cadaveric (*n* = 5) and OA (lateral *n* = 5, medial *n* = 4) specimens stained with Picrosirius Red and imaged at low (4x, top, scale bars: 200 μm) or high (40x, bottom, scale bars: 100 μm) magnification using polarized light microscopy revealed differences in subchondral bone collagen organization. Collagen fibers are less organized in OA samples than control samples and in medial regions than lateral regions. **b** The distribution of collagen fiber orientation shows significant differences between all four groups. **c**, **d** Synchrotron Radiation X-ray micro-computed tomography (SRμT) of subchondral bone from the lateral and medial sides of cadaveric control and OA tibial plateaus showed a qualitative increase in mineralization, according to the colorimetric scale (800–3 200 mg HA‧cm^−3^), in 3D-reconstructed (**d**, top) and 2D high-magnification (**d**, bottom) images. The distribution of mineralization through the subchondral bone from each region (**c**) confirms the shift in the peak mineralization level in OA medial specimens (*n* = 5) compared with OA lateral (*n* = 4) specimens. OA curves were not statistically compared with medial or lateral control samples due to low sample size (*n* = 2). Lines represent the mean of all specimens in the group. *P < 0.05 by mixed model with random intercepts

In mouse models of PLR suppression and in human osteonecrotic subchondral bone, loss of lacunocanalicular area is often accompanied by collagen disorganization and hypermineralization of the bone extracellular matrix (ECM).^13,27,30^ Therefore, we evaluated the organic and mineral constituents of OA subchondral bone. Birefringent collagen fibers in OA subchondral bone showed qualitatively less alignment relative to the control tissue (Fig. 2a). Upon quantification, collagen linearity was significantly lower in OA specimens compared with control specimens on both the lateral and medial sides. Furthermore, collagen fibers were significantly less aligned on the medial side of the joint than on the lateral side in both groups (Fig. 2b).

Consistent with these site-dependent and disease-dependent patterns, hypermineralization of subchondral bone was most pronounced in specimens from the medial side of the OA tibial plateau (Fig. 2d). Medial OA specimens also portrayed a rougher subchondral surface than control specimens. Statistical analysis confirms that the distribution of mineral density is significantly shifted in medial OA samples relative to lateral OA samples, but low sample size precludes quantitative comparison with control groups (Fig. 2c). Therefore, OA is accompanied by subchondral bone collagen disorganization and is regionally associated with bone matrix hypermineralization within samples, concordant with suppressed PLR.^13,27,30^

### Reduced osteocyte expression of PLR enzymes in human OA subchondral bone

**Fig. 3:**
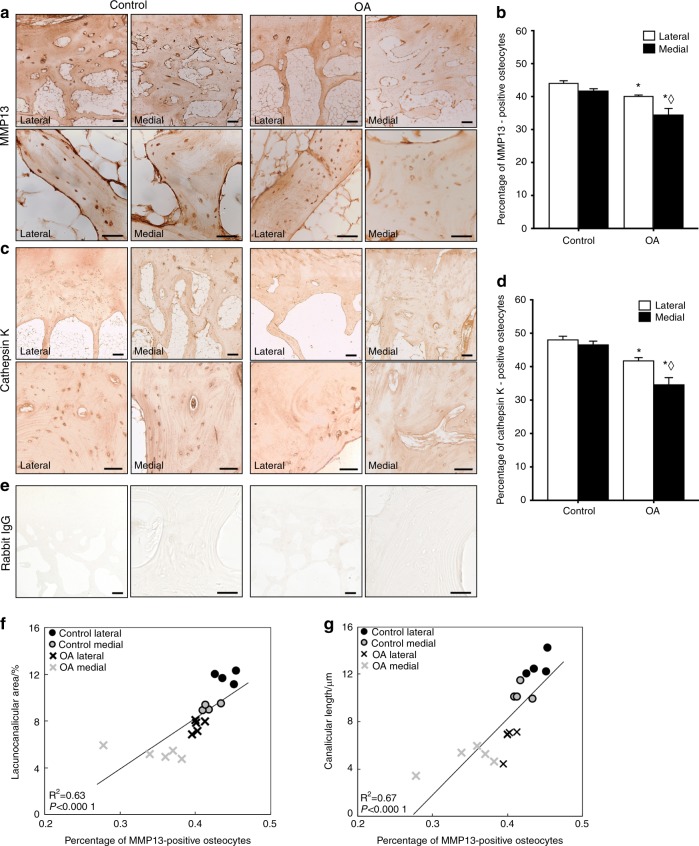
PLR enzyme expression is suppressed in human OA subchondral bone. **a**–**d** Immunohistochemical analysis of MMP13 (**a**) and Cathepsin K (CatK, **c**) levels and localization in subchondral bone from control and OA tibial plateau specimens was performed at low (4x, top, scale bars: 200 μm) and high (40x, bottom, scale bars: 100 μm) magnification. Qualitative and quantitative analyses show diminished MMP13 and CatK expression in the OA tibial plateau, with a significant reduction in the percentage of osteocytes stained positively for MMP13 (**b**, control *n* = 4, OA *n* = 5) and CatK (**d**, control *n* = 4, OA *n* = 5) in both regions. **e** No differences in negative control staining were observed. **f**–**g** Furthermore, Pearson’s product–moment correlation indicates that percent of MMP13-positive osteocytes is strongly correlated with lacunocanalicular area (**f**, *r* = 0.79, *P* < 0.000 1) and canalicular length (**g**, *r* = 0.82, *P* < 0.000 1). Graphs show mean ± SEM. *P < 0.05 compared with respective regions of control specimens, ^⋄^*P* < 0.05 between regions by Holm–Sidak post hoc tests

Given that OA subchondral bone shows multiple signs of suppressed PLR, we sought to evaluate the expression of key enzymes implicated in PLR by osteocytes. Immunohistochemistry (IHC) revealed qualitatively lower levels of MMP13 (Fig. 3a) and Cathepsin K (CatK, Fig. 3c) protein expression in subchondral bone of the medial OA tibial plateau relative to healthy controls and relative to the less severely affected lateral OA tibial plateau. Accordingly, the percentage of MMP13-positive osteocytes was lower in OA subchondral bone by 20% on the medial side and 10% on the lateral side relative to their respective control sites (Fig. 3b). The percentage of CatK-positive osteocytes was 24% lower on the medial side and 13% lower on the lateral side in OA subchondral bone compared to respective cadaveric controls (Fig. 3d). No differences in negative control immunostaining were observed between cadaveric and OA samples (Fig. 3e). Interestingly, the percentage of MMP13-positive osteocytes is strongly correlated with the lacunocanalicular area and with canalicular length for each sample and region (Fig. 3f–g).

Together these results suggest that human OA is correlated with PLR suppression in subchondral bone, as demonstrated by repression of key PLR enzymes in subchondral bone, loss of lacunocanalicular area, collagen disorganization, and hypermineralization. As a next step in evaluating the causality of PLR suppression in joint disease, we generated mice with a targeted deletion of MMP13 from osteocytes. We previously reported that systemic ablation of MMP13 suppresses PLR,^13^ however, chondrocyte expression of MMP13 contributes to cartilage degradation,^31^ and ablation of MMP13 in chondrocytes is chondroprotective.^32,33^ Therefore, we characterized the bone and joint phenotypes of mice with a novel, osteocyte-intrinsic ablation of MMP13.

### Targeted ablation of MMP13 expression in osteocytes

An established floxed MMP13 allele^34^ was deleted under control of DMP1-Cre (9.6-kb promoter),^35^ resulting in mice with a targeted deletion of MMP13 in osteocytes. DMP1-Cre^+/−^; MMP13^fl/fl^ (MMP13^ocy−/−^) animals are born at the same rate and are grossly similar to their DMP1-Cre^−/−^; MMP13^fl/fl^ (wild-type) littermates, with no significant differences in weight or lifespan.

**Fig. 4:**
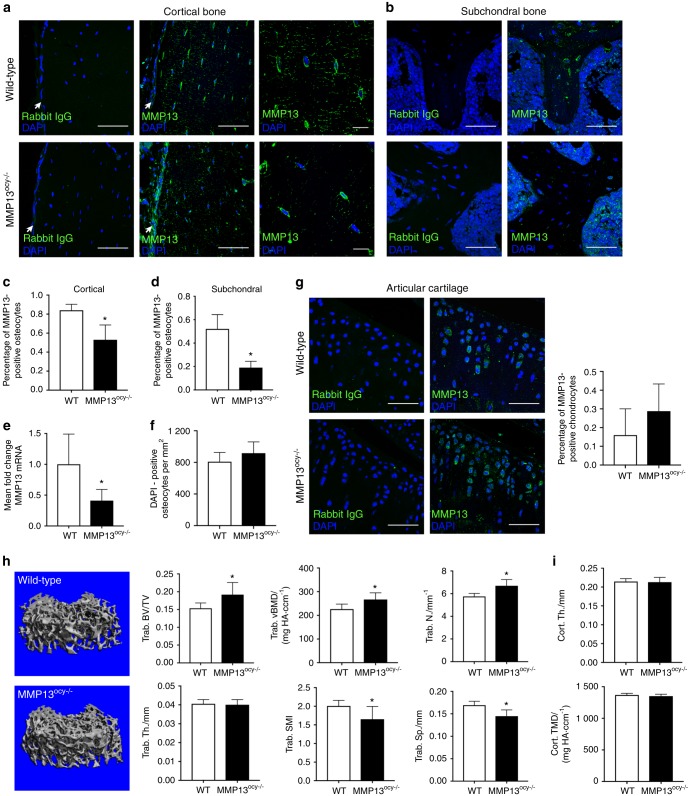
Osteocyte-specific reduction in MMP13 expression in the cortical and subchondral bone causes increased trabecular bone mass. **a**–**d** In cortical bone (**a**) imaged at low (left and center, arrows indicate periosteal surface, scale bars: 50 μm) and high (right, scale bars: 10 μm) magnification and in subchondral trabecular bone (**b**, scale bars: 50 μm), the number of osteocytes stained positive for MMP13 is reduced in MMP13^ocy−/−^ bone compared with wild-type (**c**, **d**, *n* = 6-9). No differences were observed in osteoblasts in the periosteum, in bone marrow, or in negative control images. **e** MMP13 mRNA was also significantly reduced in MMP13^ocy−/−^ bone compared with wild-type (*n* = 7–9). **f** Osteocyte number was not changed between groups. **g** MMP13 expression in chondrocytes was not significantly different between groups (scale bars: 50 μm, *n* = 6–8). **h** μCT of distal femoral trabecular bone (*n* = 11) shows that bone volume fraction and volumetric bone mineral density are increased in MMP13^ocy−/−^ bone due to an increase in the trabecular number and corresponding decrease in trabecular spacing rather than a change in trabecular thickness. Decreased SMI indicates a shift to more plate-like trabecular structures. **i** Cortical bone, however, did not present differences in thickness or mineral density. Graphs show mean ± SD. *P < 0.05 between genotypes by unpaired *t* test

We validated the tissue-specific reduction in MMP13 expression at the transcriptional and translational level. In femoral cortical bone of MMP13^ocy−/−^ animals, immunofluorescence revealed 37% fewer MMP13-positive osteocytes (Fig. 4a, c), and in subchondral trabecular bone, the number of MMP13-positive osteocytes was reduced by 63% (Fig. 4b, c, regions of interest shown in Supplementary Fig. 2a, b, channels shown separately in Supplementary Fig. 2c). This result was consistent with the 63% reduction in MMP13 mRNA expression in humeri cleaned of marrow and periosteum (Fig. 4e).

Given the goal of identifying the role of osteocyte-derived MMP13, and since the 9.6-kb DMP1-Cre promoter can induce off-target recombination in late osteoblasts and some soft tissues,^36^ we also evaluated possible changes in MMP13 expression in other cell types in the MMP13^ocy−/−^ mouse model. Immunofluorescence revealed neither significant changes in the number of MMP13-positive chondrocytes in articular cartilage (Fig. 4g) nor qualitative differences in MMP13 expression in growth plate chondrocytes in MMP13^ocy−/−^ mice (Supplementary Fig. 2d). MMP13 expression in periosteal cells (Fig. 4a), bone marrow (Fig. 4b; Supplementary Fig. 2c), and skeletal muscle (not shown) was also unchanged between genotypes. Furthermore, the number of DAPI-stained osteocytes in the cortical bone is not affected by MMP13 ablation (Fig. 4f), suggesting that recombination in this model is not affecting the differentiation and embedding of osteocytes. Therefore, the MMP13^ocy−/−^ mouse model is appropriate to observe differences in bone and joint phenotypes arising primarily from changes in osteocyte-derived MMP13.

Trabecular bone volume is increased in mice with systemic ablation of MMP13 and in other models of PLR suppression.^13,19,34^ To determine if deletion of osteocyte-intrinsic MMP13 is sufficient to alter bone mass, we used μCT to analyze trabecular and cortical bone mass and microarchitecture. Relative to wild-type mice, MMP13^ocy−/−^ femurs had a 25% increase in trabecular bone volume fraction due to a 16% increase in the trabecular number and a corresponding decrease in trabecular spacing with no change in trabecular thickness (Fig. 4h). MMP13^ocy−/−^ femurs also show an increase in volumetric bone mineral density and a decrease in SMI reflecting a shift to more plate-like microarchitecture. The mRNA levels or ratio of RANKL and OPG mRNA expression do not account for these differences (data not shown). Cortical bone thickness and total mineral density were normal in MMP13^ocy−/−^ femurs (Fig. 4i). Therefore osteocyte-intrinsic MMP13 is sufficient to alter trabecular bone volume and mineralization.

### Suppressed PLR in MMP13^ocy−/−^ bone

**Fig. 5:**
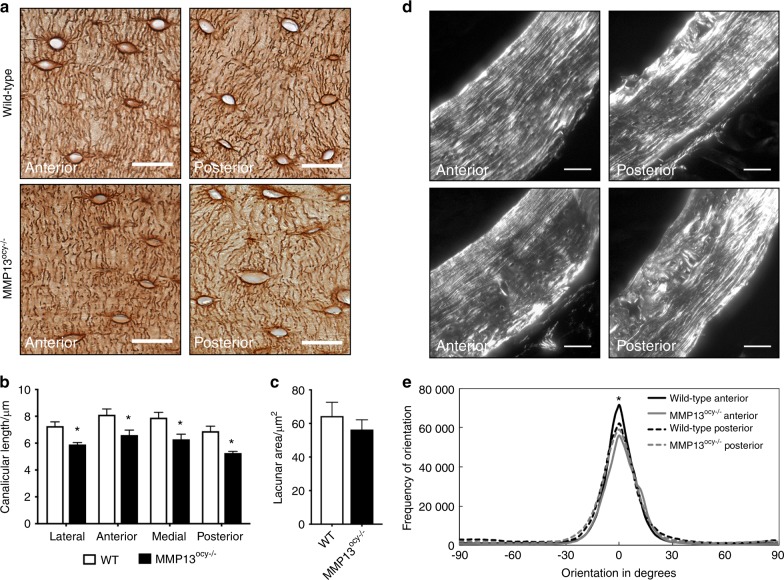
MMP13^ocy−/−^ cortical bone displays hallmarks of suppressed perilacunar/canalicular remodeling. **a**–**c** Canalicular length in wild-type bone is longer than that in MMP13^ocy−/−^ bone in all regions sampled (**b**, *n* = 7), while lacunar area is not statistically different (**c**, *n* = 7). Graphs show mean ± SEM. **d**, **e** Collagen fiber organization is reduced in MMP13^ocy−/−^ bone (*n* = 9) compared with wild-type bone (*n* = 8) in the anterior quadrant but not in other regions. Lines represent the mean of all specimens in the group. Scale bars are 20 μm in **a** and 50 μm in **d**. *P < 0.05 between genotypes by unpaired *t* test

To determine the role of osteocyte-intrinsic MMP13 in PLR, we evaluated the osteocyte LCN and collagen alignment, both of which are sensitive to PLR suppression, including in mice with systemic ablation of MMP13.^13^ The LCN of femoral cortical bone is visibly disrupted by osteocyte-intrinsic MMP13 deficiency (Fig. 5a). Canalicular length in MMP13^ocy−/−^ mice is reduced by 20% (Fig. 5b) with no significant change in lacunar area (Fig. 5c) or lacunar density (data not shown). This decrease in canalicular length occurs in a coordinated manner across the medial, lateral, anterior, and posterior regions of MMP13^ocy−/−^ cortical bone (Fig. 5b). We consistently observed a small but significant decrease in peak alignment of collagen fibers in MMP13^ocy−/−^ bone compared with wild-type bone in the anterior region (Fig. 5d, e). In the other regions studied, no differences in collagen linearity were detected despite the change in PLR activity suggested by LCN analysis.

Since changes to collagen, mineral, or LCN organization can affect bone biomechanical behavior,^13,19,37^ we tested mechanical properties of femurs from 2- and 4-month-old wild-type and MMP13^ocy−/−^ mice using three-point bending. Small but significant decreases in whole-bone structural stiffness and ultimate load were detected in 4-month-old MMP13^ocy−/−^ bones (Table 1), consistent with minor deficiencies in both collagen and mineral.^38,39^ However, no significant changes were detected in yield properties, postyield displacement, or work-to-fracture, so cortical bone biomechanical outcomes were relatively insensitive to osteocyte-intrinsic MMP13 deficiency in this model. Overall, osteocyte-intrinsic MMP13 is required for PLR since its ablation disrupts the maintenance of canalicular networks and collagen organization and reduces bone mechanical properties.

**Table 1:** Flexural properties of wild-type and MMP13^ocy−/−^ femurs

	Posterior compression, 2 months (*n* = 9)		Posterior compression, 4 months (*n* = 8–9)	
Flexural propertities	WT	MMP13^ocy−/−^	WT	MMP13^ocy−/−^
Bending stiffness/(N·mm^−1^)	83.63 ± 9.14	82.85 ± 6.00	112.24 ± 13.40	110.26 ± 9.64
Yield load/N	7.94 ± 1.71	8.07 ± 1.03	9.31 ± 1.97	11.26 ± 1.40*
Ultimate load/N	16.79 ± 2.21	15.11 ± 1.24^#^	19.02 ± 2.08	18.24 ± 1.63
Postyield displacement/mm	0.56 ± 0.22	0.60 ± 0.27	0.54 ± 0.37	0.38 ± 0.13
Work to fracture/N-mm	7.98 ± 1.87	7.86 ± 3.09	8.24 ± 3.30	6.55 ± 1.85
Bending modulus/GPa	10.97 ± 1.60	12.50 ± 2.56	15.31 ± 1.07	15.50 ± 3.26
Yield stress/MPa	107.94 ± 19.20	124.38 ± 30.27	132.45 ± 25.56	162.75 ± 32.98^#^

	Anterior compression, 2 months (*n* = 3–9)		Anterior compression, 4 months (*n* = 8–9)	
	WT	MMP13^ocy−/−^	WT	MMP13^ocy−/−^
Bending stiffness/(N·mm^−1^)	78.65 ± 10.82	67.03 ± 1.04	100.77 ± 6.70	94.25 ± 9.42
Yield load/N	8.65 ± 1.22	9.28 ± 2.29	11.08 ± 1.09	10.73 ± 1.50
Ultimate load/N	13.25 ± 1.65	13.61 ± 1.67	17.12 ± 1.34	15.64 ± 1.35*
Postyield displacement/mm	0.95 ± 0.56	1.00 ± 0.50	0.31 ± 0.10	0.37 ± 0.19
Work to fracture/N-mm	8.91 ± 2.32	9.16 ± 2.40	5.09 ± 1.50	5.27 ± 2.01

Femurs from wild-type and MMP13^ocy−/−^ mice were broken with either anterior or posterior side in compression. In general, structural and tissue material properties were stronger in posterior (physiological) compression than anterior compression and get stronger with age. Ultimate load tended to be lower in MMP13^ocy−/−^ bones than wild-type bones, particularly in 4-month-old samples broken in anterior compression. MMP13^ocy−/−^ bones also had lower stiffness in this test configuration. Values are presented as mean ± SD. *P < 0.05 between genotypes, ^#^*P* < 0.065 between genotypes by unpaired *t* test

### Increased cartilage degradation in MMP13^ocy−/−^ mice

**Fig. 6:**
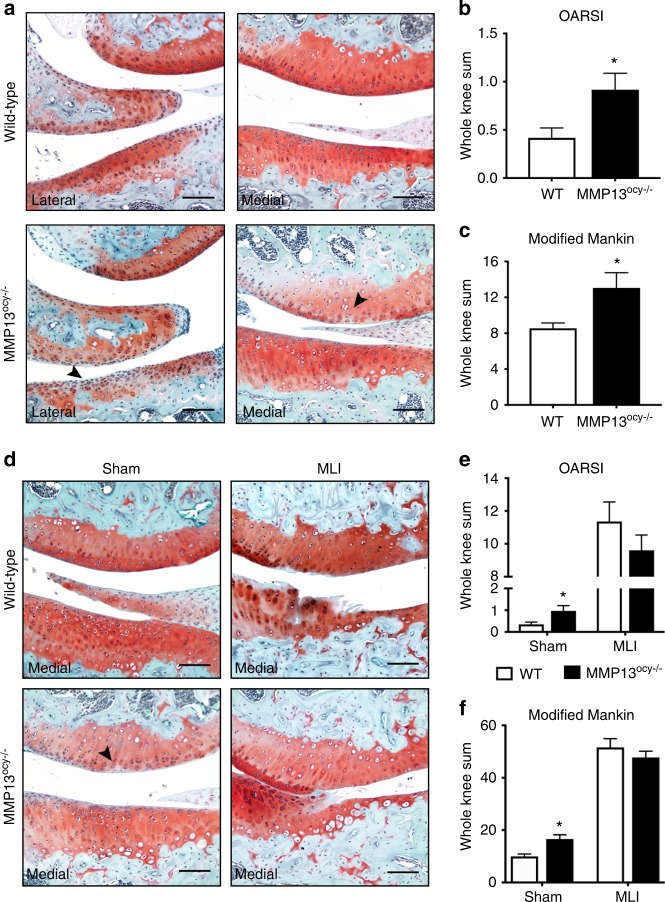
Knees of non-injured MMP13^ocy−/−^ mice show more cartilage damage than wild-type mice. **a**–**c** Safranin-O stained joints of 16-week-old wild-type mice show intact cartilage, while MMP13^ocy−/−^ mice have areas of cartilage surface irregularity and proteoglycan depletion (**a**), leading to significantly higher OARSI (**b**) and modified Mankin (**c**) scores (*n* = 6). **d**–**f** In sham-injured joints, MMP13-dependent proteoglycan loss is still apparent, and meniscal-ligamentous injury (MLI) induces severe cartilage damage in both groups (**d**). On OARSI (**e**) and modified Mankin (**f**) grading scales, sham-injured MMP13^ocy−/−^ mice score significantly higher than wild-type mice (*n* = 10), but scores are equivalent after MLI (*n* = 10 for wild-type, *n* = 11 for MMP13^ocy−/−^). Arrows denote cartilage damage in non-injured joints. Scale bars are 100 μm. Graphs show mean ± SEM. *P < 0.05 between genotypes by unpaired *t* test

Though subchondral bone clearly contributes to osteoarthritis, the role of osteocytes in joint disease remains unclear.^3,5–8^ Given the strong association of PLR suppression with cartilage degeneration in human OA (Figs. 1–3), we tested the hypothesis that PLR suppression via ablation of osteocyte MMP13 is sufficient to cause cartilage degeneration. Articular cartilage of 4-month-old wild-type and MMP13^ocy−/−^ mouse knees demonstrated clear histopathological differences (Fig. 6a). Relative to the smooth, proteoglycan-rich articular cartilage in wild-type knees, MMP13^ocy−/−^ cartilage had surface irregularities and depletion of proteoglycans. These characteristic features of degenerating articular cartilage were apparent in basal conditions on the medial and lateral tibial and femoral surfaces. Accordingly, using two established OA grading scales,^40,41^ MMP13^ocy−/−^ knees had statistically more cartilage degeneration than wild-type knees (Fig. 6b, c). Therefore, loss of MMP13 function in the subchondral bone osteocytes is sufficient to disrupt cartilage homeostasis, causing the appearance of osteoarthritic features in otherwise healthy cartilage. This result further suggests that the severe PLR suppression observed in OA human subchondral bone plays a causal role in the progression of joint disease.

To assess the role of PLR suppression in post-traumatic cartilage degeneration, we used an established medial ligamentous injury (MLI) model to induce OA in wild-type and MMP13^ocy−/−^ knees.^42^ As in non-injured controls, sham-injured MMP13^ocy−/−^ joints showed more cartilage degeneration than their wild-type counterparts (Fig. 6d). Increases in chondrocyte hypertrophy and articular cartilage lesions, as well as proteoglycan loss, contributed to the more severe OA grade in sham-injured MMP13^ocy−/−^ knees (Fig. 6e, f). After injury, both genotypes experienced severe loss of articular cartilage and osteophyte formation, but no differences were found between genotypes (Fig. 6d–f). These data suggest that although MMP13 expression by osteocytes is important for cartilage homeostasis, the severity of joint injury overshadows the osteocytic contribution to healthy joint crosstalk in this model.

### Mechanisms of subchondral osteocyte influence on cartilage

Although a causal role for osteocytes in cartilage degeneration, to our knowledge, has not previously been demonstrated, there are multiple hypothetical mechanisms by which subchondral bone deterioration could exacerbate cartilage degeneration and drive OA.^3,5,6^ These include biological hypotheses such as cell death or vascular changes, and mechanical hypotheses such as rod and plate distribution in subchondral bone.^8,43–45^ To further understand why MMP13^ocy−/−^ mice are predisposed to cartilage degeneration, we evaluated biological and structural features of tibial cartilage and subchondral bone in healthy and injured joints.

**Fig. 7:**
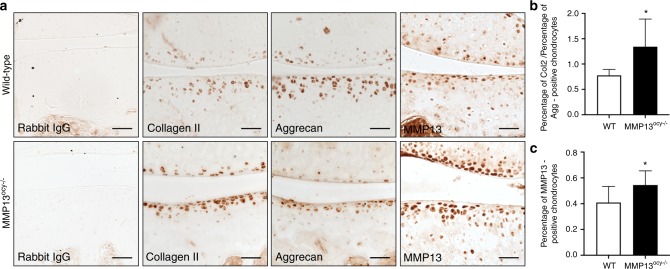
Chondrocytes in MMP13^ocy−/−^ mice have altered cartilage matrix synthesis and MMP13 expression. **a** IHC of collagen II, aggrecan, and MMP13 in wild-type and MMP13^ocy−/−^ mice (sham group) show qualitative genotype-dependent changes in cartilage matrix synthesis and degradation, with apparent increases in collagen II and MMP13 and a decrease in aggrecan. **b** The collagen II to aggrecan ratio in MMP13^ocy−/−^ chondrocytes is significantly increased (*n* = 6). **c** The percentage of MMP13-positive chondrocytes in MMP13^ocy−/−^ knees is significantly increased (*n* = 10). Scale bars are 50 μm. Graphs show mean ± SD. *P < 0.05 between genotypes by unpaired *t* test

First, we tested the hypothesis that MMP13^ocy−/−^ bone causes increased chondrocyte catabolism and apoptosis by evaluating the expression of cartilage matrix constituents collagen II and aggrecan, of degeneration markers collagen X and MMP13, and of the products of cartilage matrix degradation, the neoepitopes VDIPEN and NITEGE. Relative to the wild-type, MMP13^ocy−/−^ chondrocytes had increased levels of collagen II and a slight decrease in aggrecan (Fig. 7a). The resulting increase in the collagen II to aggrecan ratio in MMP13^ocy−/−^ cartilage (Fig. 7b) is a hallmark of early OA.^46^ MMP13 expression by MMP13^ocy−/−^ chondrocytes was also significantly elevated (Fig. 7a, c), consistent with the observed cartilage degeneration (Fig. 6). Significant differences were not observed in collagen X, VDIPEN, or NITEGE expression (not shown), nor were osteocyte or chondrocyte viability altered by osteocyte MMP13 deficiency or injury (Supplementary Fig. 3), though additional timepoints may be required to observe these cellular responses.^47,48^

**Fig. 8:**
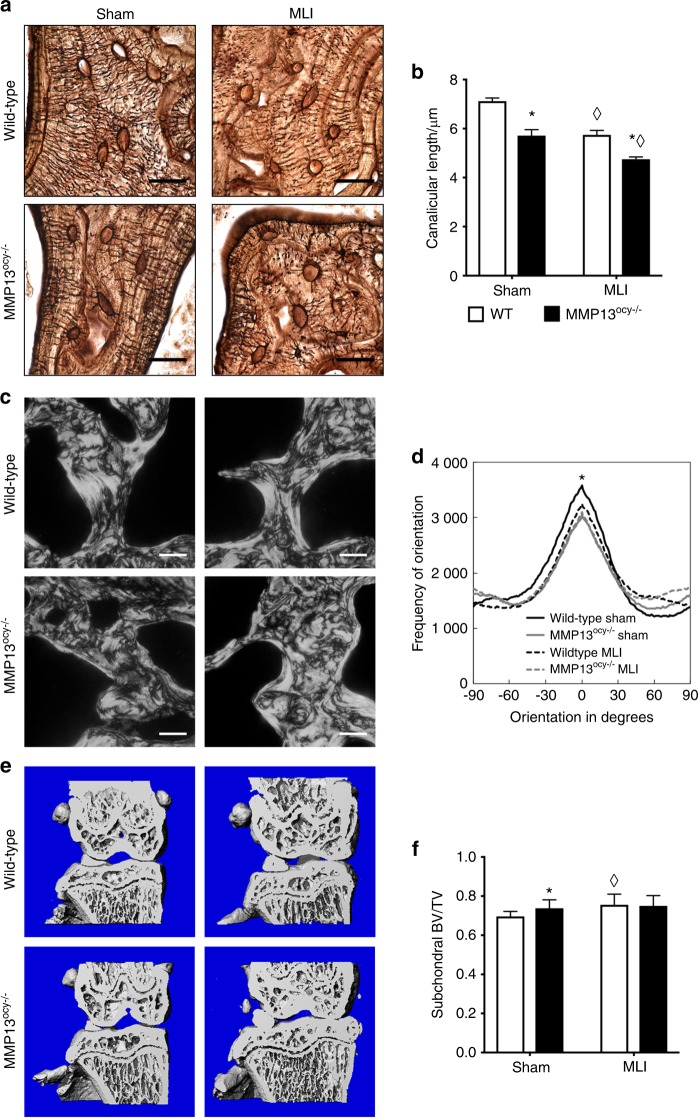
Subchondral bone shows MMP13-dependent changes before injury. **a**–**b** Like in cortical bone, canalicular length in MMP13^ocy−/−^ subchondral tibias is shorter than in wild-type subchondral tibias (**a**). The difference is statistically significant in injured and non-injured joints (**b**, *n* = 6, mean ± SEM). **c**, **d** Collagen fiber organization is significantly affected by MMP13 in sham-injured subchondral tibias, but no difference is apparent after injury (*n* = 6, lines represent the mean of all specimens in the group). **e**, **f** Similarly, subchondral bone volume fraction is increased in sham-injured MMP13^ocy−/−^ tibias (*n* = 10) compared with sham-injured wild-type tibias (*n* = 10), but after injury wild-type (*n* = 10) and MMP13^ocy−/−^ (*n* = 11) joints are indistinguishable. Graph shows mean ± SD. Scale bars are 20 μm in **a** and 50 μm in **c**. *P < 0.05 between genotypes, ^⋄^*P* < 0.05 between treatments by Holm–Sidak post hoc tests

Second, since human osteoarthritic subchondral bone is characterized by sclerosis, collagen disorganization, and disrupted LCN (Figs. 1–3), we hypothesized that these structural features would be observed in MMP13^ocy−/−^ subchondral bone. As in cortical bone (Fig. 5a), the canalicular length of osteocytes in MMP13^ocy−/−^ subchondral bone was visibly shorter than in wild-type bone (Fig. 8a). Canaliculi were 17%–20% shorter in MMP13^ocy−/−^ subchondral bone compared with wild-type bone, and injured samples had 17%–20% shorter canaliculi than in the corresponding sham-injured groups for each genotype (Fig. 8b). These findings suggest that the cartilage degradation in mice lacking osteocyte MMP13 could be due to reduced PLR and highlight the sensitivity of the LCN to joint injury. Collagen fiber organization in sham-injured tibial subchondral bone was also disrupted by osteocytic MMP13 deficiency (Fig. 8c). Joint injury caused a loss of collagen fiber organization compared to wild-type shams (Fig. 8d), but no differences between genotypes were detected after injury. Similarly, μCT of tibial subchondral bone revealed a MMP13-dependent difference in bone volume fraction between the sham groups, but not between the injured groups (Fig. 8e, f). Thus, loss of osteocyte MMP13 causes structural changes to subchondral bone that mimic joint injury, possibly via its effects on PLR.

**Fig. 9:**
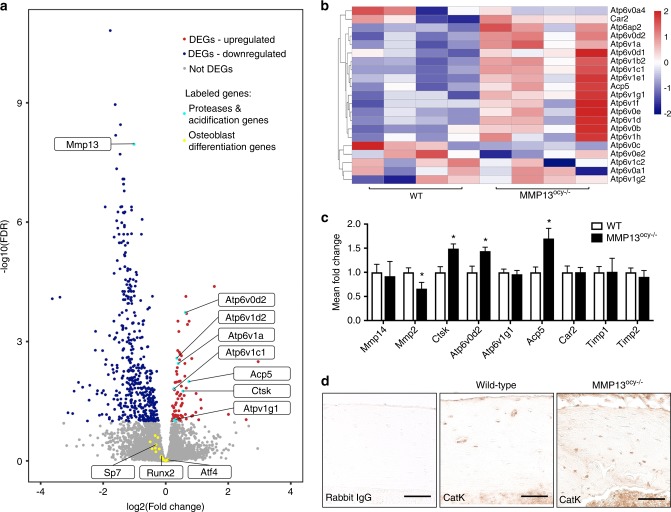
Osteocyte deficiency of MMP13 causes dysregulation of genes related to matrix degradation and acidification. **a** RNA-seq of wild-type and MMP13^ocy−/−^ mice (*n* = 4) identified differentially expressed genes (DEGs) that are significantly repressed (blue) or induced (red) in MMP13^ocy−/−^ bone, with downregulation of MMP13 and upregulation of several proteases and acidification-related genes (highlighted in turquoise). Transcription factors and other genes implicated in osteoblast differentiation (yellow) are among the genes that did not show significant expression differences in MMP13^ocy−/−^ bone (gray). **b** Genes related to acidification were hierarchically clustered in a heatmap by z-score. **c** qPCR of wild-type (*n* = 8) and MMP13^ocy−/−^ (*n* = 6) bone validates both co-regulated and compensatory expression of PLR genes (mean ± SD). **d** Immunohistochemistry reveals increased levels of Cathepsin K in osteocytes in MMP13^ocy−/−^ cortical bone (scale bars 50 μm). *P < 0.05 between genotypes by unpaired *t* test

Finally, we performed RNA-seq on wild-type and MMP13^ocy−/−^ bones to determine which biological pathways in osteocytes were disrupted by MMP13 ablation. We detected 90 upregulated and 454 downregulated differentially expressed genes in MMP13^ocy−/−^ bone, of which MMP13 was one of the most significantly downregulated (Fig. 9a). With induction or suppression of PLR, we and others have previously observed coordinated and compensatory changes in expression of genes required for matrix resorption, including proteases and genes involved in acid secretion.^19,27^ Thus, it was striking to find that the most differentially induced genes in MMP13^ocy−/−^ bone included Ctsk and Acp5, as well as many subunits of vacuolar type ATPases associated with intracellular and extracellular acidification (Fig. 9a, b). A gene signature score of acidification-related genes showed that they are significantly upregulated in MMP13^ocy−/−^ bone (*P* < 0.05). On the other hand, a gene signature score of transcription factors and markers of osteoblast differentiation showed no significant change in MMP13^ocy−/−^ bone compared with wild-type bone (*P* = 0.19), further indicating that the phenotype of the MMP13^ocy−/−^ joint is a result of defective osteocyte function rather than of osteogenic differentiation. Using qPCR, we validated that MMP13^ocy−/−^ bone has reduced expression of Mmp2 and increased expression of Ctsk, Atp6v0d2, and Acp5 (Fig. 9c). No changes were observed in Timp1, Timp2, or Mmp14. The increase in Cathepsin K is due to a change in osteocyte expression rather than osteoclast expression, which was verified by immunohistochemistry (Fig. 9d). Thus, MMP13 ablation in osteocytes not only disrupts PLR but also disrupts structural and biological homeostasis of the joint.

## Discussion

This study advances our understanding of crosstalk between cartilage and subchondral bone by implicating osteocytes as causal drivers of joint disease in osteoarthritis. In both human and mouse joints, osteoarthritic subchondral bone exhibits several outcomes of PLR suppression, including canalicular and collagen fiber disorganization, hypermineralization, and changes in PLR enzyme expression. Furthermore, osteocyte-intrinsic deficiency in the key PLR enzyme MMP13 is sufficient to suppress PLR and induce premature OA, with subchondral sclerosis and cartilage damage in otherwise healthy young mice. This study highlights a new, causal role for osteocytic MMP13 in the regulation of cartilage homeostasis and suggests PLR suppression as a novel mechanism in OA. Therefore, osteocytes emerge as a potential cellular target of new therapeutics to block or reverse OA progression.

Several features of the bone phenotype in MMP13^ocy−/−^ mice resemble those in other models of MMP13 ablation and PLR suppression. Col1-Cre^+/−^;MMP13^fl/fl^ and systemic MMP13^−/−^ mouse models together implicate bone-derived MMP13 expression in trabecular bone remodeling, PLR, and bone quality.^13,30,34^ As in systemic MMP13^−/−^ bone, osteocyte-specific MMP13 ablation increased trabecular bone mass and disrupted canalicular and collagen organization. However, whereas systemic ablation caused decreased postyield deflection and decreased fracture toughness, we observed changes to cortical bone stiffness and ultimate strength only. This was surprising because we previously observed severe defects in bending modulus, yield stress, and toughness in TβRII^ocy−/−^ bone that also has severe PLR suppression.^19^ The less severe mechanical phenotype is, however, consistent with the more subtle changes to canalicular and collagen organization in MMP13^ocy−/−^ bone. Possible explanations for these differences include the incomplete ablation of MMP13 in MMP13^ocy−/−^ bone, the contribution of non-osteocytic MMP13 to the systemic MMP13^−/−^ phenotype, partial rescue of the MMP13^ocy−/−^ phenotype by diffusion of MMP13 from cells in which it is not ablated, or the coordinated repression of multiple PLR enzymes in TβRII^ocy−/−^ but not in MMP13^ocy−/−^ bone. Whereas Mmp2, Mmp13, Mmp14, Ctsk, and Acp5 were repressed in TβRII^ocy−/−^ bone, MMP13^ocy−/−^ bone showed both co-repression (Mmp2) and an apparent compensatory upregulation (Ctsk, Acp5) of PLR enzymes. Therefore, the partial reduction of PLR enzyme expression in the MMP13^ocy−/−^ model may be insufficient to substantially impact material properties and postyield behavior measured at the whole-bone scale. Overall the MMP13^ocy−/−^ model was sufficient to suppress PLR in cortical and subchondral bone, consistent with the subchondral bone changes seen in end-stage human OA.

This MMP13^ocy−/−^ mouse model presented a unique opportunity to investigate the role of osteocytes in joint homeostasis. Since chondrocyte-derived MMP13 drives OA by cleaving collagen II,^31^ systemic MMP13 ablation is chondroprotective.^32,33^ By preserving chondrocyte expression of MMP13 while reducing its expression in osteocytes, we observed evidence of osteocyte-dependent joint crosstalk, illustrating a novel cellular mechanism by which bone affects the overlying cartilage. Because off-target expression of the 9.6-kb DMP1-Cre promoter has been reported in non-skeletal tissues and in osteoblasts,^36^ it is impossible to rule out contributions from other cell types to the observed cartilage phenotype. We anticipate that the effect on cartilage of possible non-skeletal MMP13 ablation, which participates in wound healing and cell migration,^49^ would be most pronounced in sham or MLI animals. However, the cartilage phenotype in non-injured controls and sham-operated animals was consistent, and MLI surgery was not affected by genotype. Our immunostaining and RNA-seq results suggest that loss of MMP13 in osteocytes, rather than in chondrocytes or osteoblasts, is predominantly responsible for the MMP13^ocy−/−^ cartilage phenotype. Indeed, MMP13 was not ablated in MMP13^ocy−/−^ articular cartilage, but rather was elevated, along with other outcomes of cartilage degeneration.

Together the results of this study address an important gap in understanding OA, namely the identification of mechanisms responsible for the coupled degeneration of cartilage and subchondral bone. We show here that suppression of osteocytic MMP13 causally contributes to the pathogenesis of OA, and several lines of evidence suggest that this is a PLR-dependent mechanism. First, MMP13 plays a key role in the induction and suppression of PLR. In a systemic model of MMP13 ablation and in glucocorticoid treatment, MMP13 expression in osteocytes is reduced and canaliculi are short and disorganized.^13,27^ In lactation, MMP13 is strongly upregulated as lacunar area and canalicular size increase.^10,18^ In human subchondral bone, we show that MMP13 expression is tightly correlated with LCN area and canalicular length (Fig. 3), and while the LCN phenotype is more subtle in MMP13^ocy−/−^ subchondral bone than in humans, the relationship remains. Second, several groups have observed changes to osteocyte morphology in OA subchondral bone consistent with suppressed PLR.^50–53^ Our findings complement and extend beyond these prior reports of altered osteocyte morphology, viability, and gene expression in human OA subchondral bone^50–53^ by describing the profound effect of OA on bone extracellular matrix organization, canalicular length, and lacunocanalicular network area. Furthermore, the most severe PLR suppression was evident in the medial compartment with the most cartilage damage. Finally, in mouse cortical bone, glucocorticoids repress MMP13 and other PLR enzymes, resulting in canalicular degeneration, collagen disorganization, and hypermineralization of subchondral bone,^27^ all of which mimic the signs of PLR suppression in the subchondral bone from another human joint disease, glucocorticoid-induced osteonecrosis.^25,26,54^ Nevertheless, it is possible that osteocytic MMP13 supports joint homeostasis through PLR-independent mechanisms, such as activation of latent growth factors^55–57^ or control of osteocyte differentiation or embedding. Although RNA-seq analysis indicates that MMP13^ocy−/−^ bone expresses normal levels of osteogenic differentiation markers, including Runx2, Sp7, and Atf4, an inducible model of MMP13 ablation would be needed to definitively rule out this possibility. The current findings, in which cartilage degeneration results from osteocyte-intrinsic ablation of PLR enzyme MMP13, strengthen the idea that osteocytes play a causal role in joint disease through PLR.

Much remains to be elucidated about the relative role of PLR in age-related joint degeneration and in post-traumatic OA (PTOA). Most animal models of OA involve a joint injury in which both bone and cartilage are affected, and which may additionally initiate inflammatory cascades,^40,42,58–62^ making it difficult to isolate the cell type responsible for cartilage degeneration. In our hands, MLI joint injury reduced canalicular length in both genotypes but did not affect MMP13^ocy−/−^ cartilage more severely. This suggests that reduced subchondral bone MMP13 expression is sufficient to disrupt PLR and cartilage homeostasis, but that joint injury can override preexisting defects.^63^ In human bone, the more dramatic differences in the lacunocanalicular network may be due to a combination of biochemical and mechanical effects over many years that were not replicated in our injury model. Review of additional timepoints post injury, or use of a milder injury model, may provide a clearer illustration of how osteocytic MMP13 and PLR contribute to PTOA pathogenesis. Basal phenotypes may be more representative of age-related joint degeneration, in which cartilage degeneration occurs without a known traumatic injury. The predisposition of mice with suppressed PLR to develop cartilage wear is consistent with the idea that subchondral bone sclerosis leads to cartilage breakdown. For example, in a guinea pig model of spontaneous OA, trabecular rod loss and plate thickening precede significant cartilage degradation.^8^ Likewise, in non-human primates that spontaneously develop OA, subchondral bone thickening appears to precede cartilage fibrillation.^64^ In humans, longitudinal tracking of bone marrow lesions by MRI reveals the clinical relationship of subchondral changes to OA progression and knee pain.^44^ Although similar end-stage OA phenotypes can arise from age, injury, or other causes,^65^ PLR may play a causal role in some of these pathological mechanisms, but not others. The role of PLR in OA in female patients and mice must also be considered separately since PLR, like OA, may have sexually dimorphic effects.^66^

Unraveling the circumstances and mechanisms through which a bone-intrinsic defect in MMP13 suppresses PLR and induces OA will require further studies. PLR has the potential to affect subchondral bone vascularity, microarchitecture, and mechanics, cartilage strain distribution, and cell-cell signaling, any of which could impact bone–cartilage crosstalk. The LCN, maintained by PLR, is important for solute transport, and transport between subchondral bone and cartilage is altered in mouse and human OA.^21,67,68^ Changes in subchondral bone volume fraction^43,69^ and geometry,^8^ similar to those seen in MMP13^ocy−/−^ bone, can precede cartilage pathology, possibly by changing strain distribution in the overlying cartilage. Based on our RNA-seq analyses, biological mechanisms might include autocrine or paracrine effects resulting from excessive acidification by osteocytes. If the critical points at which osteocytes cause and respond to cartilage degradation can be identified, these cells may emerge as a potential target of new therapies to prevent or treat OA. Although many questions remain about the role and regulation of PLR in OA, our data collectively suggest that osteocyte MMP13 dynamically maintains subchondral bone homeostasis and joint crosstalk, and that its disruption can exacerbate joint disease, likely through suppression of PLR.

## Materials and methods

### Human donor population and specimen preparation

Five male subjects with clinically diagnosed stage IV osteoarthritis of the tibial plateau, who were scheduled for total knee arthroplasty, were recruited for this study (Supplementary Fig. 1a). Recruitment occurred through referral from orthopedic surgeons at a Department of Veterans Affairs Medical Center. All samples were collected from patients with OA of the femorotibial joint as described in protocols that were reviewed and approved by our Human Subjects Protection Program Institutional Review Board. Informed consent was obtained from each study participant prior to enrollment. Five freshly harvested cadaveric human tibial plateaus from age-matched and gender-matched donors without history of OA, osteonecrosis, osteoporosis, or fractures were collected through the Willed Body Program at University of California, San Francisco for use as controls. Patients with OA and healthy cadaveric controls had similar BMI. All samples used for immunohistochemistry and mineralization analysis were harvested within 4 days postmortem to minimize effects of degradation. Integrity of the tissue and epitopes in histological analyses (Figs. 1, 3; Supplementary Fig. 1j, k) suggests that refrigerated storage of cadaveric samples for up to 4 days did not significantly affect the conclusions.

Each tibial plateau was removed en bloc (Supplementary Fig. 1c, d), and X-rays were collected to evaluate the severity of subchondral bone deterioration (Supplementary Fig. 1g–i). To facilitate comparison of the subchondral bone between the lateral and medial side of the joint, which was more severely affected by OA in these samples, each specimen was cut into 8–10 mm thick coronal slabs with a band saw (Supplementary Fig. 1e, f). Subchondral bone was compared between the medial and lateral regions of interest on the same osteoarthritic tibial plateau, as well as with comparable regions of cadaveric tibial plateaus. The data were collected from five samples per group for all outcomes unless otherwise specified in figure legends.

### Mice

To test the role of osteocytic MMP13 in bone and joint health, we generated mice with osteocyte-specific ablation of MMP13. Homozygous MMP13^fl/fl^ mice on an FVB background have loxP sites flanking exons 3, 4, and 5, which encode the enzyme’s active site (Jackson Laboratories #005710).^34^ Hemizygous DMP1-Cre^+/−^ mice (9.6-kb promoter) on a C57BL/6 background express Cre predominantly in osteocytes and odontoblasts (Jackson Laboratories #023047).^35^ Mice were bred at UCSF to generate wild-type (DMP1-Cre^−/−^; MMP13^fl/fl^) and MMP13^ocy−/−^ (DMP1-Cre^+/−^; MMP13^fl/fl^) mice with a mixed background. Littermate controls were used throughout. Animals were housed in groups in a specific pathogen-free environment with temperature maintained between 68 °F and 74 °F, humidity maintained between 30% and 70%, 12-h light/dark cycles, and access to water and rodent chow (LabDiet 5053) ad libitum. To match the VA population of human patient samples and to exclude the sexually dimorphic effects of both OA and PLR,^66,70^ only male mice were utilized for this study. The procedures for animal experiments were approved by the Institutional Animal Care and Use Committee at the University of California, San Francisco. Six to eleven biological replicates were used for each outcome, with exact *n* given in figure legends.

### Histology

Human tibial plateaus were fixed in 10% neutral buffered formalin (NBF) and incubated in 10% disodium and tetrasodium EDTA for 56–60 days until fully decalcified, or in an Ion Exchange Decalcification Unit (American Master Technologies) for 5–6 days, followed by serial ethanol dehydrations and paraffin embedding. Paraffin sections (7-μm thick) in the coronal plane were generated for polarized light microscopy, Safranin-O with Fast Green stain, Ploton silver stain, and immunohistochemistry. To standardize evaluation, a consistent region of subchondral bone was selected for evaluation in the medial and lateral areas of each specimen (Fig. 1a). For each specimen, values were collected from five high-powered field images per region of interest. Within each region, these values were averaged to obtain a mean value for each specimen. Each quantitative average represents an average across all specimens.

Intact mouse knee joints and proximal femurs were fixed in 10% NBF and decalcified for 2 weeks in EDTA, followed by serial ethanol dehydration and paraffin embedding. Knees were embedded at 90° of flexion and sectioned in the frontal plane. Femora were embedded and sectioned axially to generate 6-μm sections. All brightfield imaging was conducted on a Nikon Eclipse E800 microscope.

### Safranin-O/Fast Green stain and OA scoring

Safranin-O with Fast Green was used to visualize the cartilage quality of the tibial plateaus using a protocol adapted from University of Rochester.^71^ Briefly, sections were deparaffinized, rehydrated, and incubated in Weigert’s Iron Hematoxylin for 3 min. Stained slides were then washed in water and differentiated in 1% acid–alcohol for 15 s. Slides were then stained with a 0.02% aqueous Fast Green solution for 5 min and differentiated with 1% acetic acid for 30 s. Slides were then washed with water and stained in a 1% Safranin-O solution for 10 min and subsequently dehydrated, cleared, and mounted.

For human tibial plateaus, standardized OARSI grading^72,73^ was used to assess OA in Safranin-O-stained histological sections by two orthopedic surgeons. For murine samples, Safranin-O staining was conducted on sections of the knee in a plane where the ACL and PCL were visible to maintain constant region of interest. Each quadrant of the knee (medial and lateral tibia and femur) was graded by three blinded graders using OARSI^41^ and modified Mankin^40^ scales. For each sample, the numerical scores of all graders were averaged to obtain a mean score. Mean scores were then averaged within each group.

### Analysis of collagen fiber orientation by picrosirius red stain

Polarized light microscopy was performed on deparaffinized sections stained in a saturated aqueous solution of picric acid with 0.1% Direct Red-80, as described^30,74^ to visualize collagen fiber orientation. During microscopy, polarized filters were rotated to achieve the maximum birefringence before capturing each image. Red channel images were processed using the OrientationJ plug in for ImageJ as described.^75^ Statistical analysis was performed as described below.

### Analysis of the lacunocanalicular network by Ploton silver stain

To visualize the osteocyte lacunocanalicular network, sections were deparaffinized and incubated in two parts 50% silver nitrate and one part 1% formic acid in 2% gelatin solution for 55 min, as described.^30,76^ Stained slides were then washed in 5% sodium thiosulfate for 10 min and subsequently dehydrated, cleared, and mounted. Images were acquired at 100x magnification for analysis. For human tibial plateau subchondral bone, quantification of the lacunocanalicular area was performed with ImageJ by thresholding gray-scale images for dark, silver-stained lacunae and canaliculi. The resulting area was normalized to the total bone area in each image captured. Canalicular length was analyzed with ImageJ by individually measuring canaliculi surrounding osteocytes (average ten canaliculi per osteocyte). For murine cortical bone, two images were acquired in each quadrant of an axial section (medial, lateral, anterior, and posterior). In murine tibial plateau subchondral bone, four images were captured on the medial and lateral side of the joint. In each of the eight images, area of all visible lacunae was calculated with a custom, commercially available StrataQuest application (TissueGnostics), yielding approximately 90 measurements per animal. In addition, ten canaliculi on three osteocytes per image (24 osteocytes per animal) were traced using ImageJ to calculate mean canalicular length. To determine whether we had sampled canalicular length from enough osteocytes, we randomly sampled from within the 24 osteocyte averages per animal and found that group averages consistently stabilize once 15 osteocytes per animal are included in the analysis.

### Analysis of PLR enzyme expression by immunohistochemistry (IHC)

IHC was used to examine protein localization qualitatively and semi-quantitatively (i.e., % positively stained cells). For IHC, slides were deparaffinized and hydrated prior to incubation in Innovex Uni-Trieve low temperature retrieval solution (NB325) in a 40 °C water bath for 24 hours (human) or in a 65 °C water bath for 30 min (mouse). Endogenous peroxidase activity was quenched using 3% H_2_O_2_ for 10 min at room temperature. For the subsequent steps, Innovex Universal Animal Immunohistochemistry Kit (329ANK) was utilized. Samples were blocked with Fc-Block and Background Buster for 45 min each at room temperature. Primary antibodies were diluted in PBS (anti-MMP13, 1:100, ab39012; anti-CatK, 1:50, ab19027; anti-Aggrecan, 1:200, ab216965; anti-Collagen II, 1:200, ab34712; anti-CGGFVDIPEN, 1:200 and anti-CGGNITEGE, 1:200, both gifts from Dr. John Mort) and incubated in a humid chamber at 4 °C overnight. Secondary linking antibody and HRP-enzyme were both used at room temperature for 10 min each. Fresh DAB solution was applied and incubated at room temperature for 5 min prior to washing with tap water and mounting with Innovex Advantage aqueous mounting medium. Negative controls were performed by substituting rabbit IgG at the same concentration as primary antibody. Quantification was performed with the help of ImageJ Cell Counter plug in to determine the average percentage of positively stained osteocytes or chondrocytes relative to the total number of cells in each 40x magnified visual field.

In murine cortical bone, subchondral bone, and cartilage, MMP13 expression was additionally visualized and quantified using immunofluorescence. Sections were deparaffinized, and antigens were retrieved with Uni-Trieve solution as above. Sections were blocked with Background Buster (Innovex) for 10 min or 10% normal goat serum for 1 h, incubated with PBS/0.1% Tween for 5 min, and then incubated overnight with rabbit anti-MMP13 antibody (1:50). After washes in PBS, secondary goat anti-rabbit antibody conjugated to Alexa Fluor 594 (1:1 000, ab150080, pseudocolored green in Fig. 4) was applied for 60 min. Background was reduced with copper sulfate for 10 min, and slides were mounted with Prolong Gold antifade reagent with DAPI. Images were acquired on a Leica DMi8 confocal microscope. The percentage of MMP13-expressing osteocytes and chondrocytes was calculated relative to the total number of cells in at least two 40x fields per sample for 6–9 mice per genotype.

### Analysis of cell death by TUNEL assay

To detect osteocyte and chondrocyte death, mouse knee sections were deparaffinized and permeabilized in 0.1% sodium citrate with 0.1% Triton X-100 for 8 min. For a positive control, two sections were treated with DNase for 10 min at room temperature to induce DNA strand breaks. Then all samples were incubated with TUNEL reaction mix for 60 min at 37 °C (Roche). After washing, slides were mounted with Prolong Gold antifade reagent with DAPI and imaged on a Leica DMi8 confocal microscope. The total number of labeled osteocytes and chondrocytes per bone or cartilage area was calculated in the medial and lateral compartments of the tibia for six knees per group.

### Synchrotron radiation X-ray computed micro-tomography (SRμT)

To visualize and quantify bone mineralization, 4-mm-wide specimens of cartilage and subchondral bone were imaged by SRμT at beamline 8.3.2 of the Advanced Light Source (ALS) (Lawrence Berkeley National Laboratory, Berkeley) as described.^27^ Briefly, transmission radiographs were taken over a 180° rotation with a monochromatic energy of 20 keV and an exposure time of 800 ms. Computational reconstruction of 3D images reveals bone microstructure at 1.3 μm/per pixel (5X lens, LuAG scintillator). Images were segmented using ImageJ by binarization of the bone volume morphology. 3D visualization and quantification of bone mineral density was performed using Avizo (Visualization Sciences Group). The data were collected from *n* = 2 control medial, *n* = 2 control lateral, *n* = 4 OA medial, and *n* = 5 OA lateral human tibial plateaus. Statistical analysis was performed only between medial and lateral regions of OA subchondral bone, as described below.

### Micro-computed tomography (μCT)

For skeletal phenotyping, femurs were harvested from male mice at 13 weeks old and stored in 70% ethanol. Cortical analysis was conducted in a 1-mm region equidistant from the proximal and distal ends of the bone. Trabecular analysis was conducted in a 2-mm region immediately proximal to the distal growth plate. For subchondral bone analyses, knee joints were harvested from 16-week-old males and stored in saline solution at −20 °C. A 4-mm region centered on the joint was scanned. Medial and lateral tibial subchondral bone were delineated 200 μm from the proximal surface of the tibia and extended for 250 μm distally. The medial and lateral femoral condyles were designated 200 μm from the distal end of the femur and extended proximally 200 μm. All samples were scanned using a Scanco μCT50 specimen scanner with an X-ray potential of 55 kVp, current of 109 μA, and voxel size of 10 μm. Thresholding and quantification were performed as previously described.^19,27^

### RNA-seq and quantitative RT-PCR analysis

Humeri from wild-type and MMP13^ocy−/−^ mice were cleaned of muscle and periosteum, epiphyses were trimmed, and marrow was removed by centrifugation. Bones were snap-frozen in liquid nitrogen prior to homogenization in TRIzol (Invitrogen), as described.^19,27^ mRNA was purified using the RNeasy Mini Kit following the manufacturer’s instructions (Qiagen).

For RNA-seq, samples were sequenced on the Illumina HiSeq 4000 at the UCSF Functional Genomics Core. Single-end 50 bp RNA-seq reads were aligned to the Ensembl mouse GRCm38.87 reference genome using STAR 2.5.2b aligner. We obtained 367.4 million total reads and average of 82.6% of these reads aligned uniquely to the mouse genome. The DESeq2 package in R Statistical Computing Environment was used to find differentially expressed genes with false discovery rate of 0.1.^77^ To calculate gene signature scores, we generated gene sets of acidification-related genes (Fig. 9b) and osteoblast differentiation-related genes (Runx2, Sp7, Atf4, Alpl, Col1a1, Col1a2, Dmp1, Sost, Bglap, Bglap2, Spp1, Phex, Mepe, Enpp1, and Enpp2),^78^ subtracted the mean from each element, and divided by the standard deviation. RNA-seq data have been deposited in the NCBI BioProject: PRJNA549974.

For qPCR, 1 μg RNA per sample was reverse-transcribed using the iScript cDNA synthesis kit. qPCR was performed using Taqman probes for β-actin (assay #Mm02619580_g1) and MMP13 (assay #Mm00439491_m1 which targets exons 4-5) and using iQ SYBR Green Supermix (BioRad) with β-actin as the housekeeping gene (primer sequences given in Supplementary Table 1). In total, 20 ng equivalent of cDNA was used per reaction for β-actin, and 30 ng–50 ng equivalent of cDNA was chosen for each test gene to achieve threshold values of amplification between 20 and 30 cycles. Expression was then quantified by the ΔΔCt method.^79^

### Flexural strength tests

Whole-bone biomechanical properties were measured in femurs isolated from 2-month-old and 4-month-old wild-type and MMP13^ocy−/−^ mice. Whole hydrated femurs were loaded to failure in three-point bending using a Bose Electroforce 3200 test frame. One femur per mouse was broken in the direction of primary physiological bending (posterior compression), and the other was broken against the direction of physiological bending (anterior compression). An 8-mm span was chosen because it was approximately 50% of the bone length. Tests were performed in air at a fixed displacement rate of 10 μm‧S^−1^. Whole-bone stiffness was calculated from the linear portion of the load-displacement curve, and yield was designated as the point where a line representing a 10% loss in stiffness intersected the load-displacement curve.^80^ Following fracture, bone cross-sections were imaged by scanning electron microscopy on a Sigma 500 VP FE-SEM (Zeiss) at an excitation voltage of 15 kV and a partial pressure of 35 Pa. Measurements of cross-sectional diameter and thickness were acquired in ImageJ and used to calculate moment of inertia assuming an elliptical cross-section. These geometric parameters were used to convert the load-displacement data to stress–strain data in order to measure tissue modulus, tissue stress, and tissue strain with standard beam theory equations.^81^

### MLI surgery

Eight-week-old male mice were separated into three groups: control, sham, and meniscal-ligamentous injury (MLI).^42^ Under general isofluorane anesthesia, both hind limbs of MLI animals were shaved and sterilized. A bilateral approach was chosen in order to minimize effects of altered biomechanics arising from a single knee injury, as previously described.^82,83^ Briefly, medial incisions through the skin and joint capsule were made adjacent to the patella to expose the medial collateral ligament, which was transected. The medial meniscus was then removed. Sham-injured animals received bilateral incisions without MCL transection or meniscus dissection. Skin incisions were closed with sutures, and animals received an injection of long-acting buprenorphine analgesic. Control animals did not receive anesthesia or analgesics. All animals were allowed unrestricted activity, food, and water. At 16 weeks of age, animals were euthanized and hind limbs collected for histological and radiographic analyses.

### Statistics

Comparisons between two groups were tested with unpaired two-tailed Student’s *t* test. Comparisons between disease state and region in human specimens or between genotype and injury in mice were tested with two-way ANOVA followed by Holm–Sidak post hoc tests. Analyses were performed in GraphPad Prism 8 (GraphPad Software, Inc.). The Clinical and Translational Science Institute Statistical Consulting Service at UCSF evaluated significant differences in the distributions of human collagen organization and mineral density (SRμT) among each group using a mixed model with random intercepts. A linear model was used for the fixed effects, and the outcome was logarithmically transformed. In all figures, *P*-values < 0.05 were considered statistically significant and are reported as such. As appropriate for each outcome, the mean is shown + /− SD or SEM, as specified in figure legends.

## Supplementary information

Supplemental Material
