# Supplementary material for: Osteocyte dysfunction promotes osteoarthritis through MMP13-dependent suppression of subchondral bone homeostasis
Source: Bone Res. 2019 Nov 5;7:34. doi: 10.1038/s41413-019-0070-y (PMC6828661; doi:10.1038/s41413-019-0070-y)
Supplement: Supplementary file 1 — Supplemental Material [file 41413_2019_70_MOESM1_ESM.docx]

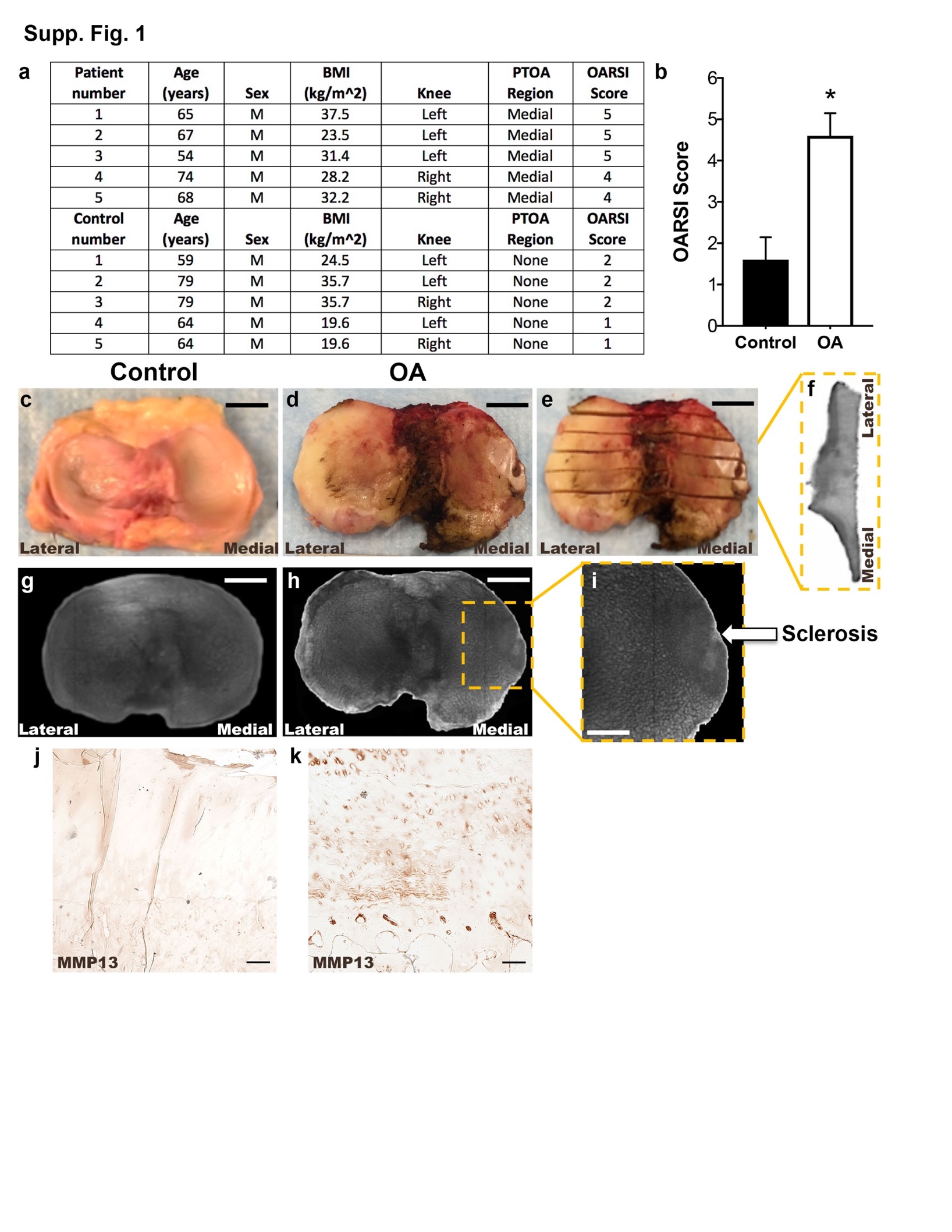


**Supplemental Figure 1:** Osteoarthritic human tibial plateaus have cartilage degradation and subchondral bone sclerosis compared to healthy controls. **a-i** Human tibial plateau specimens from patients with osteoarthritis (OA) undergoing total knee replacement surgery were compared to control cadaveric human tibial plateaus from donors with no history of OA, osteonecrosis, osteoporosis, or fractures. Donations were controlled for age, sex, and body mass index (BMI) (a). OARSI scores (b) were consistent with gross (c, d) and radiographic (g, h) evidence of OA in surgical specimens. Gross cartilage degradation and sclerosis predominated in the medial compartment of OA specimens (i). For further histological analysis, specimens were cut into six 8-10 mm thick coronal slabs (e, f). **j-k** While control specimens (j) could not be fixed as quickly as OA specimens (k), immunohistochemical analysis of MMP13 suggests that this delay caused neither increased protease expression nor significant epitope degradation, since MMP13 expression in control samples is qualitatively lower in chondrocytes, but higher in subchondral osteocytes (Figure 3a-b) compared to in OA specimens. Scale bars are 2 cm in c-h, 1 cm in i, and 200 μm in j-k. Graph shows mean ± SD. *p<0.05 between groups by unpaired t-test.


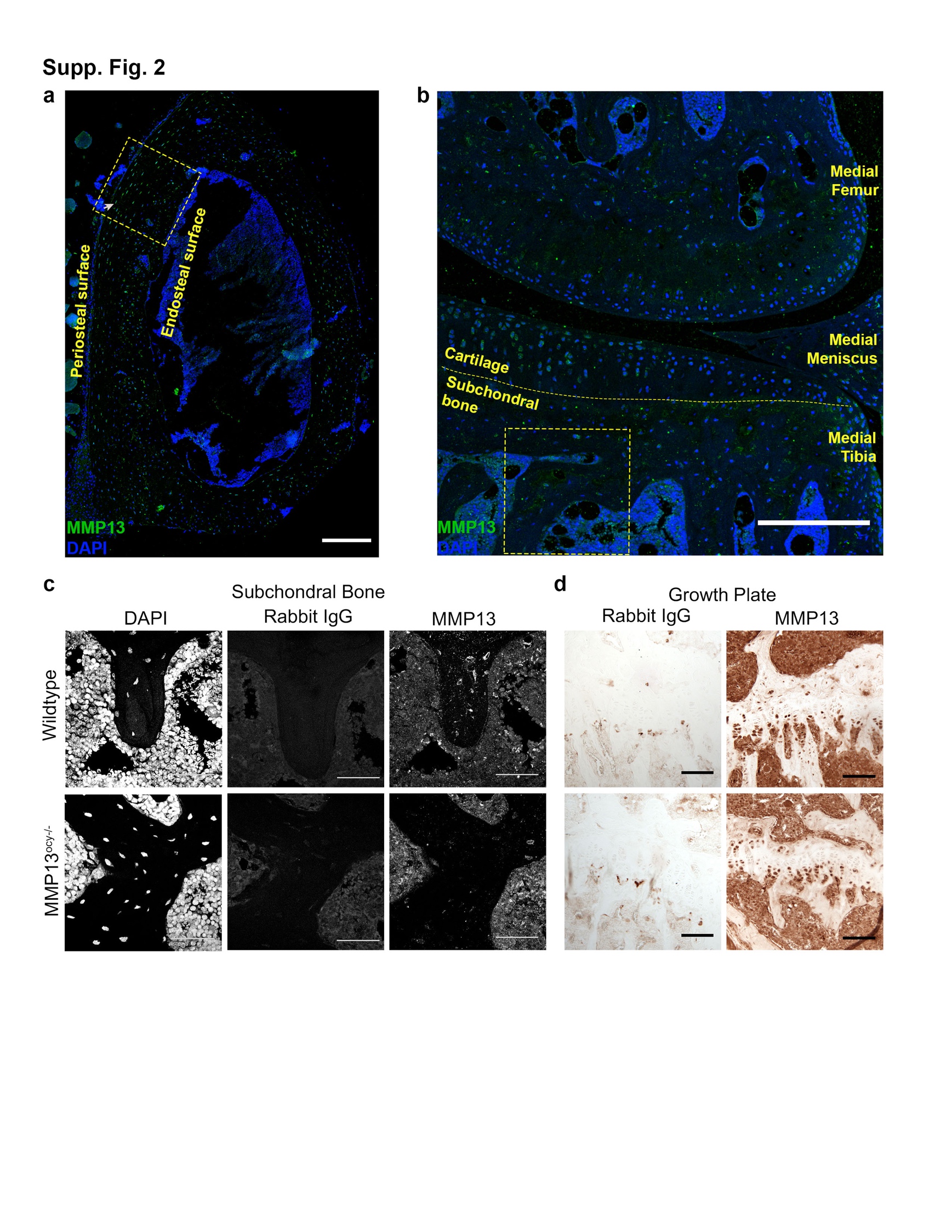


**Supplemental Figure 2:** Data supporting phenotyping of MMP13^ocy-/-^ mice. **a** The yellow box on the axial section of murine femur demonstrates a representative region of interest for cortical bone images in Figure 4a (arrow indicates periosteal surface). **b** The medial femur, tibia, and meniscus are labelled in a frontal section of the murine knee. The yellow box demonstrates a representative region of interest for subchondral bone images in Figure 4b. Scale bars 200 μm. **c** Both immunofluorescence and immunohistochemistry were used to evaluate the efficiency and specificity of MMP13 ablation in osteocytes of MMP13^ocy-/-^ mice. To better visualize this difference, DAPI staining is shown separately from MMP13 and negative control immunofluorescence corresponding with Figure 4b. Scale bars 50 μm. **d** MMP13 expression in growth plate chondrocytes is not significantly different between genotypes. Scale bars 100 μm.


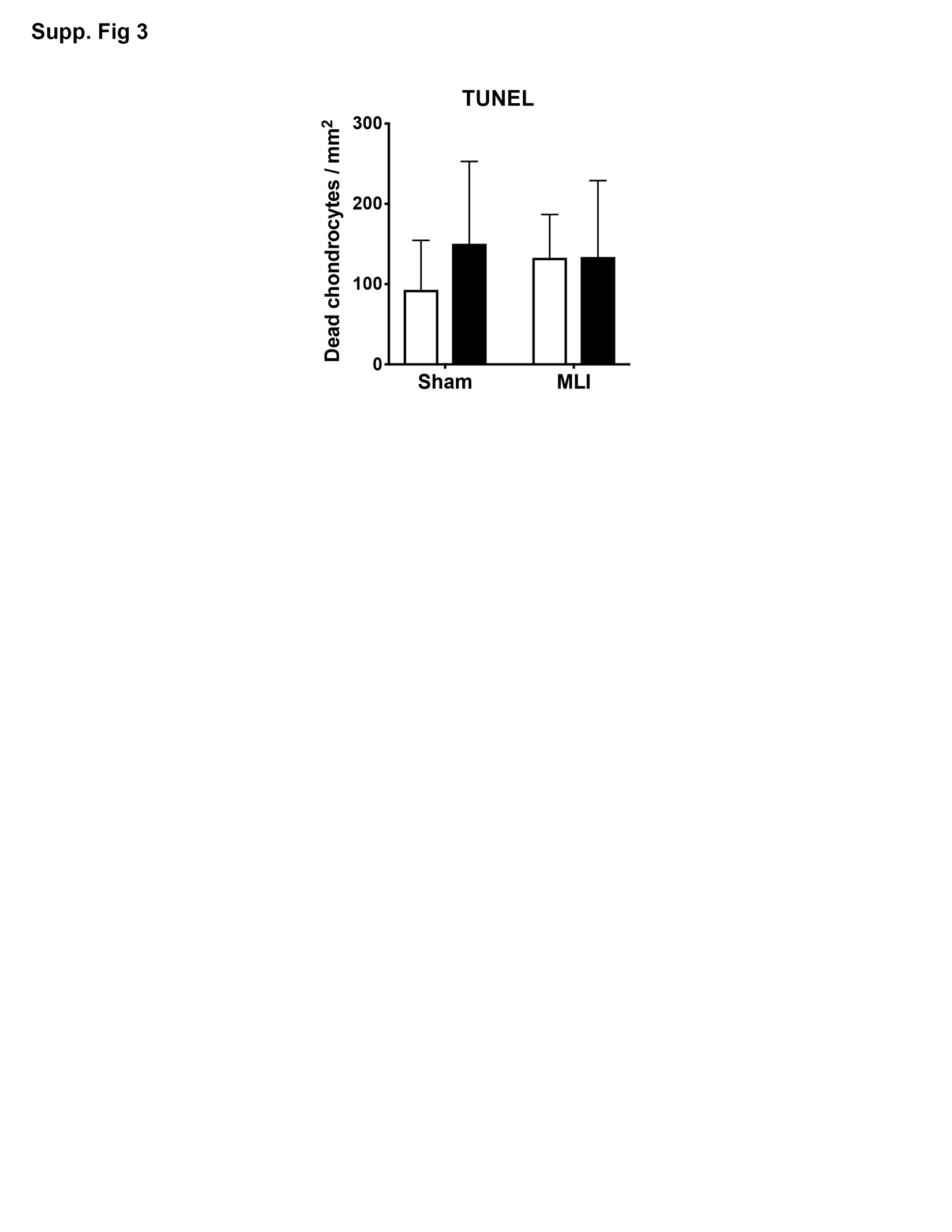


**Supplemental Figure 3:** No difference was detected in TUNEL-positive chondrocytes due to genotype

or treatment (n=6).

**Supp Table 1**

| **Gene** | **Sequence** |
| --- | --- |
| b-actin (sense)  b-actin (antisense) | 5’-CTCTGGCTCCTAGCACCATGAAGA-3’  5’-GTAAAACGCAGCTCAGTAACAGTCCG-3’ |
| Mmp13 (sense)  Mmp13 (antisense) | 5’-CGGGAATCCTGAAGAAGTCTACA-3’  5’-CTAAGCCAAAGAAAGATTGCATTTC-3’ |
| Mmp14 (sense)  Mmp14 (antisense) | 5’-AGGAGACGGAGGTGATCATCATTG-3’  5’-GTCCCATGGCGTCTGAAGA-3’ |
| Mmp2 (sense)  Mmp2 (antisense) | 5’-AACGGTCGGGAATACAGCAG-3’  5’-GTAAACAAGGCTTCATGGGG-3’ |
| Ctsk (sense)  Ctsk (antisense) | 5’- GAGGGCCAACTCAAGAAGAA-3’  5’- GCCGTGGCGTTATACATACA-3’ |
| Acp5 (sense)  Acp5 (antisense) | 5’-CGTCTCTGCACAGATTGCAT-3’  5’-AAGCGCAAACGGTAGTAAGG-3’ |
| Rankl (sense)  Rankl (antisense) | 5’-CCAAGATCTCTAACATGACG-3’  5’-CACCATCAGCTGAAGATAGT-3’ |
| Opg (sense)  Opg (antisense) | 5’-AGAGCAAACCTTCCAGCTGC-3’  5’-CTGCTCTGTGGTGAGGTTCG-3’ |
| Timp1 (sense)  Timp1 (antisense) | 5’-CTCAAGACCTATAGTGCTGGC-3’  5’-CAAAGTGACGGCTCTGGTAG-3’ |
| Timp2 (sense)  Timp2 (antisense) | 5’-AGTGCAAGATCACTCGCTGT-3’  5’-CGCGCAAGAACCATCACTTC-3’ |
| Car2 (sense)  Car2 (antisense) | 5’-GAGCTTCACTTGGTTCACTGG-3’  5’-TGTGAGGCAGGTCCAATCTTC-3’ |
| Atp6v1g1 (sense)  Atp6v1g1 (antisense) | 5’-TGAGCAGTTGACTTAGGCCG-3’  5’-CCTCCGGTTCTTTCGCTTGC-3’ |
| Atp6v0d2 (sense)  Atp6v0d2 (antisense) | 5’-TCTTGAGTTTGAGGCCGACAG-3’  5’-GCAACCCCTCTGGATAGAGC-3’ |

**Supplemental Table 1:** SYBR primer sequences used for gene expression analysis of murine mRNA.
